# Workplace Bullying in Early Childhood Education Settings: Prevalence and Protective Factors

**DOI:** 10.1007/s13158-022-00341-y

**Published:** 2022-10-31

**Authors:** Laura McFarland, Rebecca Bull, Tamara Cumming, Sandie Wong

**Affiliations:** 1 Melbourne Graduate School of Education, University of Melbourne, Melbourne, VIC Australia; 2 Macquarie School of Education, Macquarie University, Sydney, NSW Australia

**Keywords:** Workplace bullying, Early childhood education and care, Early childhood workforce, Early childhood educators, Horizontal violence, Educator well-being

## Abstract

Workplace bullying in the early childhood education and care (ECEC) sector is a pervasive and significant issue in Australia and globally. Workplace bullying can negatively impact early childhood professionals’ mental health, contributing to staff turnover and attrition. Given the current, and predicted, future shortages of ECEC staff, it is critical that strategies be implemented to support staff well-being and maintain healthy and safe workplaces. The aims of this study were to examine the current prevalence of workplace bullying in the ECEC sector in Australia and to identify protective workplace factors associated with lower prevalence of workplace bullying. Using a convergent parallel mixed methods design, findings are drawn from qualitative and quantitative survey questions within a large study on ECEC educators’ well-being conducted in Australia. Participants were 591 early childhood professionals working in ECEC a variety of ECEC settings. Findings indicated that 24.6% of respondents reported experiencing workplace bullying and that most bullying was perpetrated by co-workers. Some workplace factors were related to lower rates of bullying, including positive teamwork, better supervisor relations, lower work-related stressors and having greater influence on workplace decisions. Implications of the findings are discussed in relation to informing policy and practices to address workplace bullying in the ECEC sector by identifying aspects of the workplace that serve as protective factors.

## Résumé

L'intimidation au travail dans le secteur de l'éducation et de la garde des jeunes enfants (EAJE) est un problème omniprésent et important en Australie et dans le monde. L'intimidation au travail peut avoir un impact négatif sur la santé mentale des professionnels de la petite enfance, contribuant au roulement et à l'attrition du personnel. Compte tenu des pénuries actuelles et prévues de personnel d'EAJE, il est essentiel que des stratégies soient mises en oeuvre pour soutenir le bien-être du personnel et maintenir des lieux de travail sains et sûrs. Les objectifs de cette étude étaient d'examiner la prévalence actuelle de l'intimidation au travail dans le secteur de l'EAJE en Australie et d'identifier les facteurs de protection sur le lieu de travail associés à une prévalence plus faible de l'intimidation au travail. En utilisant une conception de méthodes mixtes parallèles convergentes, les résultats sont tirés de questions d'enquête qualitatives et quantitatives dans le cadre d'une vaste étude sur le bienêtre des éducateurs de l'EAJE menée en Australie. Les participants étaient 591 professionnels de la petite enfance travaillant dans divers contextes d'EAJE. Les résultats ont indiqué que 24,6 % des répondants ont déclaré avoir été victimes d'intimidation au travail et que la plupart des intimidations étaient perpétrées par des collègues. Certains facteurs liés au lieu de travail étaient liés à des taux plus faibles d'intimidation, notamment un travail d'équipe positif, de meilleures relations avec les superviseurs, des facteurs de stress liés au travail moins élevés et une plus grande influence sur les décisions en milieu de travail. Les implications des résultats sont discutées par rapport à la politique et aux pratiques d'information pour lutter contre l'intimidation au travail dans le secteur de l'EAJE en identifiant les aspects du lieu de travail qui servent de facteurs de protection.

## Resumen

El acoso laboral en el sector de la educación y el cuidado infantil es un problema generalizado y de gran importancia Australia y alrededor del mundo. El acoso laboral puede tener un impacto negativo en la salud mental de los profesionales de la primera infancia, lo que contribuye al abandono de la profesión y al desgaste del personal. Dada la escasez actualy futura prevista de personal en el nivel infantil, es fundamental que se diseñen e implementen estrategias para apoyar su bienestar y mantener lugares de trabajo saludables y seguros. Los objetivos de este estudio fueron examinar la prevalencia actual del acoso laboral en el nivel infantil en Australia e identificar factores de protección asociados con una menor prevalencia del acoso laboral. Basándonos en un diseño mixto convergente y paralelo, los datos se obtuvieron de preguntas de encuestas cualitativas y cuantitativas, enmarcadas en un estudio a gran escala sobre el bienestar de los educadores de infantil en Australia. Los participantes fueron 591 profesionales de la primera infancia que trabajan en una variedad de entornos. Encontramos que el 24,6% de los encuestados informaron haber experimentado acoso en el lugar de trabajo y que la mayoría de los acosos fueron perpetrados por compañeros de trabajo. Algunos factores de protección se relacionaron con bajas tasas de intimidación, incluido el trabajo en equipo positivo, mejores relaciones con los supervisores, menos factores estresantes relacionados con el trabajo y mayor capacidad de influencia en las decisiones laborales. Las implicaciones de los hallazgos se discuten para informar políticas y prácticas que permitan abordar el acoso laboral en contextos de educación infantil, mediante la identificación de aspectos laborales que sirvan como factores clave de protección.

## Introduction

Many people will experience workplace bullying at some point in their life, which can have negative long-term physical and emotional consequences (Ludlow, 2021). In Australia, one in three women and one in five men who have claimed a mental health disorder acquired in the workplace, state that harassment and bullying was a contributing factor (Safe Work Australia, 2020). Worryingly, 37% of workers across a range of sectors report having been harassed at the workplace (Safe Work Australia, 2020). Research indicates that nearly half of Australian employees have experienced workplace bullying at some stage—a figure that is similar in Europe and North America (Butterworth et al., 2015).

The early childhood education and care (ECEC) sector is in the top three professions for occurrence of workplace bullying (Lucas, 2018). According to a report from Employsure, a large provider of workplace relations and health and safety services in Australia, 40% of people working in the ECEC sector in Australia reported experiencing workplace bullying (Morgan, 2018). Only those working in farming and hospitality reported more workplace bullying than ECEC (Morgan, 2018). Despite ECEC being a profession based on caring, it appears that this may not extend to staff, and that workplace bullying is pervasive (Ludlow, 2021).

Given that workplace bullying can negatively impact educators’ mental health and contribute to a toxic work climate, educator–child interaction quality may suffer as a result (Aboagye et al., 2021). Bullying may also contribute to the high staff turnover and attrition rate currently evident in Australian ECEC (Thorpe et al., 2020). This is problematic, as the sector is facing widespread staff shortages now and, in the future (O’Connell, 2019). In order to create supportive and inclusive work environments to help redress staff turnover and attrition, it is important to better understand aspects of the workplace which protect against bullying. The current study examines three research questions. First, what is the prevalence and source of workplace bullying in ECEC. Second, are there educator or centre characteristics that are more likely than expected to be associated with bullying? Finally, what workplace factors are associated with a lower prevalence of bullying. We begin by providing some context to situate the current study in the Australian ECEC sector. Next, we review the current literature about the prevalence and impact of workplace bullying, as well as the workplace factors that are associated with workplace bullying. We then present the current study, the methodology, and findings. Results are drawn from the survey component of a large study into ECEC educators’ well-being conducted in Australia by the (De-identified). Conclusions and implications of the findings are then discussed in relation to informing policy and practices to address workplace bullying in the ECEC sector by identifying aspects of the workplace that serve as protective factors.

## Context of the Current Study

The current study included ECEC educators from a range of early childhood service types, including long day care (services providing care and education to children in the years prior to school which operate full-day), preschool (services that provide education to children aged 3–5), occasional care (services offering care to children on an as-needed basis), and family day care (services providing care and education to children in the home of a qualified educator) (Victoria Government, 2021). In Australia, there are a range of educational roles, including educators with at least a diploma level qualification, educators with a certificate III level qualification, and Early Childhood Teachers (ECTs), with a degree level qualification (Australian Children’s Education & Care Quality Authority [ACECQA], n.d. a). ECTs hold the leadership role in the classroom. Adult child ratios are required in family day care, and in long day care and preschools. Ratios differ depending on the age of the children: birth-24 months (1:4), over 24 months–up to 36 months (1:5), over 36 months (1:10 or 1:11 depending on the state (ACECQA, n.d. b.).

ACECQA is an independent national authority that assists the Australian Government to implement the National Quality Framework (NQF). The NQF provides a national approach to regulation, assessment, and quality improvement for ECEC services (ACECQA, 2017). Services in Australia are assessed and rated by ACECQA based on seven National Quality Standards (NQSs): educational program and practice, children’s health and safety, physical environment, staffing arrangements, relationships with children, collaborative partnerships with families and communities, and governance and leadership (ACECQA, 2017). Services are then given a rating against the NQS (exceeding, meeting, working toward, significant improvement needed). Services rated as “exceeding” in all quality areas (QAs) and who “promote exceptional education and care and demonstrate sector leadership, and commitment to continual improvement” can apply for “excellent” classification through a separate process.

## Literature Review

### Workplace Bullying

In Australia, workplace bullying is defined by the Australian Government as occurring when “a person or group of people repeatedly behave unreasonably towards another worker or group of workers”, resulting in “a risk to health and safety” (Fair Work Ombudsman, n.d., n.p.). Examples can include aggressive behaviour towards others, teasing, pressuring someone to engage in inappropriate behaviour, excluding others from work-related activities, or setting unreasonable or unachievable work demands. Research from 1528 Australian employees found that the most common forms of work-related bullying involve having information withheld and being ordered to do work below one’s level of competence (Magee et al., 2014). Given the pervasiveness of bullying in the workforce at large, it is important to identify workplace factors associated with bullying.

Little data are available about the incidence of workplace bullying in ECEC settings, making it difficult to understand the scope and particulars of the reasons for bullying, as well as to design preventative strategies. An analysis of workers’ compensation data from 2016–2017 in one Australian state (New South Wales, Australia’s most populous state) showed that 39% of claims for psychological injury were for work-related harassment and/or bullying. (Cumming et al., 2020a). However, data on sources of the bullying, or breakdowns between harassment and bullying, were not available. This example demonstrates the way that researchers must currently piece together and infer information in the absence of reliable, specific data.

Some studies have evidenced experiences of bullying in the ECEC sector. Morgan, (2018), for example, found that staff reported experiencing a variety of bullying acts, such as being belittled in front of families or other staff, receiving warnings without justification, and direct harassment and harassment through social media. Similarly, an Australian study of “dark side” leadership practices reported examples of educators being belittled, having duties and access to resources removed and obvious exclusion from conversations (Brooker and Cumming, 2019).

Another form of interpersonal workplace bullying, “horizontal violence”, occurs from peer to peer, rather than from the top down, and is common in fields such as nursing and ECEC (Hard, 2006). Horizontal violence involves repeated and continuous psychological harassment, such as “verbal abuse, threats, intimidation, humiliation, excessive criticism, innuendo, exclusion, denial of access to opportunity, disinterest, discouragement and the withholding of information” (McKenna et al., 2003, p.92). From her qualitative study with 26 educators working in Australian ECEC settings, Hard (2006) reported a “lingering discourse of niceness and a culture which condones behaviours that marginalise and exclude others. The outcome of this culture is a powerful expectation of compliance which does little to foster or encourage leadership activity” (p. 40). These experiences suggest that bullying can occur in explicit or tacit ways, operating horizontally between educators as well as from the top down.

In ECEC, bullying occurs regardless of work role (e.g. director, educator, assistant), and the source of bullying can vary (e.g. staff, families) (Ludlow, 2021). However, little is known about differing experiences of bullying across roles or other demographic factors. Based on data from a 2019 nationally representative survey of workplace inclusion in Australia (Brown et al., 2020), Gide et al., (2022) reported that discrimination and harassment are disproportionately experienced by minority groups. These groups include Aboriginal and Torres Strait Islander peoples, people with disabilities and those from non-Christian backgrounds. While data recording the number of people working in ECEC and belonging to one or more of these groups are not available (Gide et al., 2022), qualitative evidence of toxic workplace experiences suggest that bullying based on perceived “difference” occurs in ECEC. For example, Authors reported exclusionary behaviour against an educator because of the way they smelled to others. This educator was also a South African person of colour and employed as a casual (rather than permanent) staff member, working in a group of staff with little ethnic diversity. This example suggests that bullying can target those with multiple perceived differences.

More recently, data from the same survey that informs the current study showed a gradient of the perceived acceptability of different aspects of participants’ identity. When participants were asked to indicate their strength of agreement–disagreement with how welcome aspects of their identity were in the workplace, they agreed or strongly agreed that their cultural identity (74%) and sexual identity were the most welcome (75.3%), but only 63% reported that their religious/spiritual identity was welcome. Workplace bullying in ECEC is clearly a complex issue. Without reliable, specific data on bullying, however, change will continue to be difficult to address.

### Impact of Workplace Bullying

The negative effects of workplace bullying can be significant, leaving individuals with physical and/or emotional trauma and causing serious disruptions in organisational culture (Ludlow, 2021). On an individual level, workplace bullying can negatively affect self-confidence and self-esteem (Randle, 2003) and physical health, such as increased blood pressure, more gastrointestinal upset, and more frequent headaches (see Sauer & McCoy, 2017), and lead to poor mental health and burnout (Laschinger et al., 2010). Psychologically, workplace bullying can lead to increased anxiety, depression, and feelings of stress (Laschinger et al., 2010). Additionally, when educators’ well-being is compromised, the quality of care and education they deliver may suffer, negatively affecting the children with whom they work (Henry et al., 2021).

In addition to the devastating physical and psychological impacts that individuals may experience because of workplace bullying, organisations can also suffer. Bullying is estimated to cost employers between A$17,000 and A$24,000 per case, and the Australian economy up to $36 billion per year (House of Representatives Standing Committee on Education and Employment [HRSCEE], 2012). Other costs include loss of productivity and morale, and reputational damage (HRSCEE, 2012), as well as increased staff absences and turnover (Brooker and Cumming, 2019).

Staff turnover and attrition are also disruptive for children and families (National Scientific Council on the Developing Child, 2015). Given the potential negative effects of workplace bullying on individuals, children, families and ECEC organisations—and the future of the ECEC workforce—it is critical to better understand workplace factors that can prevent bullying. As indicated by the dated nature of sources, these understandings are currently hampered by a lack of consistent and reliable data (HRSCEE, 2012) that has not improved since this observation was made over a decade ago.

### Workplace Factors and Bullying

In Australia, employers have an obligation under the work health and safety legislation in their state or territory to manage workplace bullying and ensure employees’ safety at work (Victorian Legislation, 2004). The preferred approach to managing bullying is prevention, as there is little evidence that interventions after-the-fact are effective (WorkSafe Victoria, 2021). It is also important to view bullying not as an individual interpersonal issue, but as a wider “cultural, organisational and structural issue” (Magee et al., 2014, p. 5). Thus, identifying workplace factors associated with workplace bullying is essential to developing and implementing protective strategies.

A variety of theoretical approaches have been used to explain the occurrence of workplace bullying, including frustration/strain, interpersonal conflict, and intra-group factors (Magee et al., 2014). The frustration/strain explanation suggests that bullying results from job pressure and stress, role changes and difficult management styles. Indeed, research has found that the risk of bullying is higher in environments where there are high workloads, high stress, a lack of autonomy and decision making over one’s work, and confusion about roles (Magee et al., 2014). In addition, jobs with high emotional demands often have higher levels of bullying (Potter et al., 2016).

The interpersonal conflict explanation suggests that although workplace conflict is natural, bullying occurs when this conflict is mismanaged. To support this explanation, research has found that strong leadership is critical to the prevention of bullying (Potter et al., 2016). Adequate training in conflict management for leaders, managers and staff is needed to prevent and address interpersonal conflicts (Magee et al., 2014).

Finally, the intra-group explanation suggests that workplace bullying occurs when the team and organisational climate enable it. Indeed, the most important factor in preventing bullying is a supportive and positive workplace culture, with strong leadership, that does not tolerate bullying (WorkSafe Victoria, 2021). Effective leaders and managers are essential to creating a positive workplace culture and establishing appropriate policies, standards, and behaviours (WorkSafe Victoria, 2021).

## Methodology

### Research Design and Rationale

Data for this study were drawn from the online survey component of a larger study on ECEC educators’ well-being conducted in Australia by the (De-identified). A convergent parallel mixed methodology was used to elicit qualitative and quantitative data. In convergent parallel mixed methodology, qualitative and quantitative data are collected at the same stage of the study, analysed separately, and interpreted together (Crewell & Plano Clark, 2011). This methodology was used to provide a comprehensive and nuanced understanding of workplace bullying in the ECEC sector. Closed survey questions were used to describe the sample and gain summary data of participants’ experiences of workplace bullying (as described in the measures section), and to gather demographic data. Open-ended survey questions provided opportunities for participants to expand on their experiences of workplace bullying. Direct quotes from participants will be used to support and expand on the quantitative findings.

### Participants

There were 880 survey responses received. Respondents who provided basic demographic information but who did not complete any of the measures or answer the question about bullying were removed from the dataset (*n* = 138). Of the participants who had data for at least one measure (*n* = 742), 591 responded to a question about bullying. Initial analyses examined differences between respondents and non-respondents to the workplace bullying question; there were no differences on any of the workplace predictors (all *p*s > 0.10). For purposes of continued analysis, we report descriptive data only for participants who responded to the bullying question (*n* = 591, 97.6% females; mean age = 44.43, SD = 10.52; 12 reported being Aboriginal or Torres Strait Islander). Additional participant demographics are shown in Table 1.

**Table 1:** Participant and workplace demographics

Variable	Valid *n*	Valid %
*Qualification*		
No formal qualification	6	1.0
Certificate (minimum qualification to work in ECE in Australia; assisting the delivery of an approved learning framework)	41	6.9
Diploma (qualified to lead and leading and contribute to service delivery that meets the National Quality Standards)	225	38.1
Bachelor’s Degree (teaching degree; leading the delivery of National Quality Standard requirements)	295	49.9
Higher than Bachelor’s Degree	24	4.1
*Total years of experience*		
Less than 3 years	12	2.1
3–5 years	54	9.3
6–10 years	95	16.3
11–15 years	78	13.4
16–20 years	96	16.5
More than 20 years	247	42.4
*Experience in current workplace*		
< 1 year	62	10.6
1–2 years	67	11.4
3–5 years	145	24.7
6–10 years	133	22.7
11–15 years	74	12.6
16–20 years	45	7.7
> 20 years	60	10.2
*Service type*		
Long day care	356	60.2
Preschool	216	36.5
Occasional care	4	0.7
Family day care	2	0.3
Other	13	2.2
*ACECQA rating*		
Excellent	29	5.0
Exceeding	267	46.4
Meeting	242	42.0
Working towards	38	6.6
*Work status*		
Permanent full time	358	60.8
Permanent part time	195	33.1
Casual	16	2.7
Other	20	3.4
*Position*		
Director	354	60.3
Teacher	85	14.5
Educator	77	13.1
Room leader	32	5.5
Assistant	23	3.9
*Age of children*		
Under 2 years	27	4.6
2–3 years	30	5.1
3–5 years	284	48.6
Mixed ages	243	41.6
*Location*		
Urban	366	62.5
Semi-rural	109	18.6
Rural or remote	111	18.9

### Procedure

In March 2021, each service listed on the Australian Children’s Education and Care Quality Authority (ACECQA) database was contacted about the study. The survey link was initially emailed to service directors/managers who then distributed the survey information and link to the educators in their service. Due to the voluntary nature of the study, service directors/managers could choose to distribute the survey to educators. Because the survey results were anonymous, it is unknown how many service directors/managers distributed the survey. This study was approved by the researchers’ university Human Research Ethics Committee and was conducted in accordance with the Australian National Statement on Ethical Conduct in Human Research (National Health & Medical Research Council, 2018).

### Measures

The online survey included items from the Early Childhood Educator Well-being Survey (ECEWS) (Cumming et al., 2020b). The ECEWS contains items derived from standardised instruments and additional demographic and work-context questions developed by researchers from extant literature. The ECEWS includes demographic questions based on those used in two other studies: selected questions from the *You Bet I Care!* (YBIC) survey (Centre for Families, Work, and Well-being, 2000); and questions from The Exemplary Early Childhood Educators at Work Project (Press et al., 2020). For the current analysis, only items addressing participant and centre demographics and work environment were included, with the purpose of addressing the second research question related to workplace factors that are predictive of bullying.

#### Teamwork and Organisational Climate

We used two scales from the Work Environment Scales of the Work Health Check (WHC, Gadinger et al., 2012) to capture teamwork and positivity of the organisational climate. The scale “teamwork” (TW, 5 items) measured individual assets and perceptions as well as organisationally shared norms of support and collective action for mutual benefits (e.g. “*people support each other when problems arise*”). The scale “positive organisational climate” (POC, 6 items) integrated the extent to which employees’ input for improving existing work conditions was valued by management, the quality of leader–subordinate relationships, and employees’ commitment to the organisation’s mission (e.g. “*the management values our suggestions for improvement*”). Note that due to an administration error, POC item 4 was omitted (“*our supervisor supports us in difficult situations*”). Participants responded on a 5-point scale from strongly disagree (1) to strongly agree (5). In the current sample, reliability was good for both scales (TW Cronbach’s *α* = 0.93, POC Cronbach’s *α* = 0.80).

#### Co-worker Relations

We used seven questions from the YBIC survey which asked the respondent to indicate all phrases that describe their relationship with their co-workers most of the time. Example phrases included “*I enjoy the company of my colleagues*” and “*my colleagues are not very helpful*”. The dependent measure was the count of positive responses; an indication of all four positive statements but none of the three negative statements would result in a maximum score of 7. The scale showed marginal reliability (Cronbach’s *α* = 0.65).

#### Supervisor Relations

We used nine questions from the YBIC survey which asked the respondent to indicate all phrases that describe their relationship with the person who supervises them. Example phrases included “*supervises me too closely*” and “*sets high but realistic standards*”*.* The dependent measure was the count of positive responses; an indication of all five positive statements but none of the four negative statements would result in a maximum score of 9. The scale showed acceptable reliability (Cronbach’s *α* = 0.76).

#### Autonomy/Decision Making

We used seven items from the YBIC survey which aimed to capture how much influence the respondent has in common organisational decisions and actions. Example items included “*ordering materials and supplies*” and “*planning daily schedule of activities*”. We included four additional items focused on influence regarding what shifts are worked, who is in the work team, when holidays can be taken and work coverage for staff absences. For all items, responses were provided on a 3-point scale (very little influence, some influence, considerable influence), with higher scores indicating greater influence on decision making. The scale showed good reliability in the current sample (Cronbach’s *α* = 0.87). We also used an additional scale from the YBIC survey to examine how workplace decisions are made. Respondents were asked to indicate all items that apply to how decisions are made in their centre most of the time. Example phrases included “*people provide input but the decisions have already been made*” and “*teachers make decisions about things that directly affect them*”. The dependent measure was the count of positive responses, an indication of all four positive statements, but none of the four negative statements would result in a maximum score of 8. The scale showed acceptable reliability (Cronbach’s *α* = 0.78).

#### Identity Acceptance

Respondents were asked three questions about the acceptance of their cultural, sexual and religious/spiritual identity in their work environment (e.g. “*my cultural identity is welcome in my work environment*”). Responses were given on a 5-point scale (1 = strongly disagree, 5 = strongly agree). The scale showed good reliability (Cronbach’s *α* = 0.91).

#### Workload Stressors

We used the Work-Related Stress (WRS) scale from the Teacher Stress Inventory (TSI) (Fimian, 1984). Example questions included “*the pace of the work day is too fast*” and “*there is little time to prepare for my program and/or responsibilities*”. Responses were given on a 5-point scale (1 = not noticeable, 5 = extremely noticeable). The scale showed good reliability (Cronbach’s *α* = 0.85).

#### Families and Governance

Quality area (QA) 6 of the National Quality Standards (NQS) addresses families’ relationship with the centre, for example, their involvement in service decisions, the respect of families’ expertise, culture, values and beliefs, and the support provided to families about parenting and family well-being. We anticipated that where there was evidence of high-quality relationships with families, the frequency of bullying from families would be lower. QA7 includes reference to the clear definition of roles and responsibilities and effective decision making, and the ability of leadership to build and promote a positive organisational culture and professional learning community. We anticipated that where there was evidence of higher quality in this area, there would be less frequent reporting of bullying from managers.

#### Workplace Bullying

The question about bullying was taken from the Offensive Behaviour scale from the Copenhagen Psychosocial Questionnaire (COPSOQ, Kristensen et al., 2005). Respondents were asked “*Have you been exposed to bullying in your workplace in the last 12 months?*” The definition for bullying was given, “*Bullying means that a person is repeatedly exposed to unpleasant or degrading treatment, and that the person finds it difficult to defend himself or herself against it*”. Response options were “yes, daily”, “yes, weekly”, “yes, monthly”, “yes, a few times” or “no”. If respondents indicated that they had been exposed to bullying, they were then asked to indicate by whom they had been bullied (colleagues, manager, families or other).

#### Analysis Strategy

To address the first research question, we report descriptive statistics (frequencies) and triangulate the data with direct quotes from the qualitative responses. For the second research question, associations between bullying and participant and workplace demographics (see Table 1) are examined using chi-square analyses. To address the final research question, we firstly compare bullied and non-bullied individuals on each workplace measure using Mann–Whitney tests (to account for non-normality of data distributions) and then consider which of these workplace factors uniquely predicts lower likelihood of being bullied using logistic regression analysis.

## Results

Our first objective was to examine the prevalence and source of workplace bullying in ECEC in Australia. Our second objective was to identify potential protective workplace factors that exemplify services with lower prevalence of workplace bullying.

### Prevalence and Sources of Workplace Bullying

When asked “*Have you been exposed to bullying at your workplace during the last 12 months?*”, 24.6% (*n* = 146) of participants said “yes”. Of these, 81.5% reported this occurring a few times, 7.5% said monthly, 6% said weekly and 4% said daily. When asked who the bullying came from, 50.7% said colleagues, 40.4% said manager/supervisor, 21.2% said families and 6.2% said other (e.g. competing service, government department, centre leaders being bullied by staff members). Twenty-seven participants (18.5%) reported experiencing bullying from multiple parties. Open-ended responses did not reveal examples additional to those offered as options in the quantitative response section.

Other staff members were the most common reported source of bullying. One participant expanded on her experience of bullying from the service director, illustrating the creation of a toxic work environment.

The biggest impact is workplace bullying from our Director. We are threatened daily and are verbally and emotionally abused. Our workplace has recently begun working on well-being and this is guided by our Director. She laughs about people having 'issues' and doesn't have any understanding (Participant 262).

Another participant who was a director described experiences of bullying from staff.

As a Director I have experienced bullying by one of my staff. I have felt financially threatened by this (that I will lose my job based on her false allegations) (Participant 6).

Some participants also described experiences of what could be considered horizontal violence.

Educators that I supervise sometimes exhibit a ganging type of approach to consultation in staff meetings (Participants 3).

Another participant expressed concern that horizontal violence was occurring towards staff who chose to study for higher qualifications.

Female dominated industry where uneducated women are working to bring down women who have invested in higher learning. Horizontal violence (Participant 219).

In relation to bullying from families, one participant reported the impact for staff of not having policies or practices in place to protect staff from bullying by families.

The harassment and bullying from families over fees (mainly only from a management perspective). Feeling unsupported as there are no safe guidelines in place to protect us, only our service policies which parents will already try to challenge (Participant 141).

Another participant expressed concern over the pervasive nature of bullying from families.

I believe the greatest impact on educator well-being is the disrespect, bullying, harassment we receive multiple times a day by the parents of the children in our care. This occurs in person, phone calls, and emails that target and denigrate educators (Participant 194).

### Workplace Factors and Bullying

We next examined whether workplace bullying was related to demographic characteristics of participants and services. Frequency of experiencing bullying (e.g. a few times, monthly, weekly, daily) was collapsed to create a bivariate variable of experiencing or not experiencing workplace bullying. Chi-square tests indicated no significant association between bullying and the following variables: ACECQA rating, $\chi^{{2}}$ (3) = 7.11, *p* = 0.07; service type, $\chi^{{2}}$ (4) = 3.49, *p* = 0.479; work status, $\chi^{{2}}$ (3) = 3.78, *p* = 0.287; age of children, $\chi^{{2}}$ (3) = 5.92, *p* = 0.116; location, $\chi^{{2}}$ (2) = 0.99, *p* = 0.611.

There was a significant association between workplace bullying and educator qualification ($\chi^{{2}}$ (4) = 11.75, *p* = 0.008), total years of experience ($\chi^{{2}}$ (5) = 13.26, *p* = 0.021), experience in current centre ($\chi^{{2}}$ (6) = 18.77, *p* = 0.005) and position ($\chi^{{2}}$ (5) = 11.39, *p* = 0.044). Educators with a lower qualification (certificate), those with between 6 and 10 years of experience, those who recently started at their current centre (< 2 years) and those holding the position of room leader, were more likely than expected to report that they experienced bullying.

We then considered scores on each of the workplace predictors. Table 2 shows the comparisons of the bullied and non-bullied groups. Those in the bullied group reported greater work-related stressors, more negative teamwork and co-worker relations, more negative organisational climate and supervisor relations, poorer acceptance of identity, and less autonomy in centre and everyday work decisions. The QA6 rating (families and community) was significantly higher in the bullied group, but there was no significant difference on QA7 (governance and leadership).

**Table 2:** Descriptive data (mean, SD) for workplace factors in the bullied and non-bullied groups

	Non-bullied	Bullied	Group comparison^a^	Effect size^b^
TSI WRS	3.02 (0.93)	3.38 (1.00)	U(585) = 24,982***	0.22
WHC TW	4.17 (.69)	3.58 (.88)	U(511) = 34,577***	0.41
WHC POC	4.25 (.59)	3.78 (.74)	U(587) = 44,544.5***	0.38
Co-workers^c^	5.58 (1.46)	4.48 (1.73)	U(509) = 30,217.5***	0.25
Supervisor	6.75 (1.92)	5.34 (2.59)	U(568) = 40,110***	0.31
Identity	4.17 (0.77)	3.98 (0.84)	U(583) = 35,700*	0.12
Influence	2.43 (0.48)	2.16 (0.54)	U(588) = 4153.5***	0.29
Decisions	6.50 (1.72)	5.44 (2.47)	U(585) = 39,541.5***	0.23
QA6	2.36 (0.57)	2.47 (0.61)	U(561) = 26,041*	−.10
QA7	2.52 (0.61)	2.60 (0.62)	U(560) = 26,926	−.07

*p < 0.05; ***p < 0.001

^a^All data were non-normality distributed (Shapiro–Wilk *p* < .001). Group comparisons were made with Mann–Whitney test

^b^rank biserial correlation

^c^question answered only by those working in an organisation with more than two employees (i.e. beyond just the educator and direct supervisor)

Finally, we conducted logistic regression to examine the unique predictors of bullying (not bullied = 0, bullied = 1). Analysis was conducted using Mplus V8.7 (Muthén & Muthén, 1998–2021) with MLR estimation (maximum likelihood with robust standard errors), which does not assume normality of data distribution or independence of observations. To avoid the listwise deletion of missing data, we also used integration = montecarlo. All predictors were allowed to covary. There were four significant unique predictors: higher teamwork and supervisor relations scores, greater influence in organisational decisions and action, and lower work-related stressors. These predictors were all associated with reduced likelihood of being in class 1 (bullied, see Table 3). Odds ratios are interpreted as the change in odds for a one unit change in the predictor variable; for example, a one unit change in the teamwork variable results in 55.2% (1–0.448) less likelihood of being in class 1 (bullied), while a 1 unit change in TSI work-related stressors results in 34.7% (1.347–1) increase of being in class 1 (bullied). We also ran the same analysis using listwise deletion of missing data; the pattern of results was similar except for TSI WRS, which was no longer significant (*p* = 0.165).

**Table 3:** Predictors of workplace bullying

Predictor	Estimate	Standardised estimate	*p*	Odds ratio
TSI WRS	.298	.136	.015	1.347
Teamwork	−.803	−.299	< .001	0.448
Co-workers	−.039	−.029	.657	0.962
POC	−.020	−.006	.940	0.980
Supervisor	−.178	−.187	.003	0.837
ID acceptance	.276	.105	.071	1.318
Influence	−.732	−.177	.003	0.481
Decisions	.065	.062	.339	1.067
QA6	.162	.045	.562	1.175
QA7	.219	.068	.388	1.245

## Discussion

Workplace bullying is a pervasive and problematic issue in ECEC (Lucas, 2018) and can have devastating consequences for individuals and the workforce (Aboagye et al., 2021). As one of our participants explained, bullying can create a toxic environment, “*My previous job impacted my mental health to an extent that I had to seek counselling services. The bullying was relentless and targeted (Participant 16)*”. Given the negative impact of workplace bullying on mental health and the sustainability of the ECEC workforce, this study sought to examine the current prevalence of bullying in the ECEC sector in Australia and to identify protective workplace factors associated with lower prevalence of bullying.

### How Prevalent is Workplace Bullying in ECEC?

Results of the current survey show a rate of workplace bullying (in the 12 months to March 2021) as 24.58%. In a previous implementation of the same study with 63 participants (in the 12 months to May 2020), the prevalence of bullying was 21.9%. This indicates a slight increase, but a relatively stable rate over time. Given the reported additional stressors present in the ECEC sector during the height of the COVID-19 pandemic (Eadie et al., 2021), these rates are perhaps lower than might be expected. Nevertheless, the finding that one in four educators reported being bullied in the workplace indicates that there is an issue that should be addressed.

Results from the survey also revealed more nuanced data about who is doing the bullying, with bullying most frequently being from colleagues, followed by supervisors and then families. In particular, the results stand in stark contrast to NQS 4.2.1 Professional Collaboration, which requires that “Management, educators and staff work with mutual respect and collaboratively, and challenge and learn from each other, recognising each other’s strengths and skills”, and NQS 7.2, “Effective leadership builds and promotes a positive organisational culture and professional learning community”. Further, bullying from families would make it difficult for educators and directors to address NQS 6.1 Supportive Relationships with Families, which requires that “Respectful relationships with families are developed and maintained and families are supported in their parenting role”. Notably, there was a difference on QA6, with the average quality rating of the bullied group being higher than that of the non-bullied group; there was no difference on QA7. This might suggest that the evidence required from and provided by services to demonstrate that services are at least “meeting” these NQSs is not accurate, or not representative of the real situation across staff. Further research is required to understand the reasons why families are bullying ECE staff, and action taken to provide clearer guidelines to families about expectations of respectful behaviour towards staff.

Those who reported experiencing bullying also reported greater work-related stressors, more negative teamwork and co-worker relations, more negative organisational climate and supervisor relations, poorer acceptance of identity, less influence over everyday work decisions and less positive experiences in centre decision making. These findings strongly support the intra-group explanation of workplace bullying—whereby the team and organisational climate enable bullying to occur. As Magee et al., (2014) suggest, it seems that appropriate training is required for both team members and managers in ECEC settings to prevent and redress the incidence of bullying.

### Protective Factors

Analysis of the quantitative survey data revealed a number of protective factors in relation to bullying. Positive teamwork, for example, significantly reduced the likelihood of being a recipient of bullying. Teamwork measured organisationally shared norms of support and collective action for mutual benefits (e.g. having a “we are together” attitude, cooperation, mutual support, and information sharing). In the current study, a one unit increase on this teamwork measure predicted 55.2% less chance of being in the bullied group and was the largest effect found when we compared the scores for those who had been bullied versus not bullied. Better supervisor relations also reduced the likelihood of being a recipient of bullying. Better relations are characterised by (amongst other things) supervisors who encourage innovation, show awareness of the challenges of balancing work and family responsibilities, set high but realistic standards, and are available (Bloom & Abel, 2015). Lower work-related stressors (e.g. too much administrative paperwork, class size too big, little time to prepare) were associated with a lower likelihood of being the recipient of bullying. Similarly, a greater influence in organisational decisions and action was associated with a lower likelihood of being bullied. This measure focused on actions associated with everyday work (e.g. ordering supplies, planning daily schedule and being involved in actions and decisions at the organisational level—such as developing or changing policies, hiring staff and determining work rosters). These results support the frustration/strain explanation of bullying (Magee et al., 2014) and suggest that additional attention to job design and autonomy may also contribute to the prevention of bullying in the future. Future research could also consider whether, and in what ways, intra-group and frustration/strain explanations of bullying are related.

### Limitations

The reference period of the current study was during the height of the COVID-19 pandemic in Australia (March 2020–March 2021). This might partly explain the bullying from families who were likely both stressed and fearful during this period. It is also difficult to determine the direction of causality concerning bullying; does bullying results in more negative feelings about work environment, or does a negative work environment result in more bullying? To answer this question, an intervention would need to be put in place with the aim of reducing bullying (e.g. enhancing feelings of teamwork) to see if cases of reported bullying declined.

While most measures showed evidence of good reliability, the measure of co-worker relationships showed only marginal reliability. It is possible that this measure is capturing different elements of co-worker relations, for instance those associated specifically with working relationships (“*my colleagues share ideas and resources*”) versus those associated with personal relationships (“*my colleagues share personal concerns with me*”). Future research should consider the use of an alternative measure with a clearer focus on specific types of relationships to better delineate their association with intention to leave and job commitment.

Another limitation is that due to the voluntary and anonymous nature of the study, we do not know how many directors chose to distribute the survey to educators, nor do we know the reasons that influenced their decision to distribute the survey or not. This could have implications for the generalisability of the findings.

Finally, the absence of an association between quality ratings and rates of bullying may have limited accuracy, as quality ratings are at the service level and so do not capture impacts for individual educators or directors. To accurately gauge the presence of bullying in a service, alternative measures to those currently offered by the NQF may be required.

## Conclusion and Implications

The findings from the study contribute current data on the prevalence of bullying in the ECEC sector in Australia, as well as more nuanced accounts of who is doing the bullying. Both quantitative and qualitative data suggest the need for greater attention by government, service providers, educators, and directors on improving knowledge and taking preventative actions.

A key mechanism for ensuring these preventative actions could be revising the NQF to make clearer the requirements for ensuring staff members’ psychological health and safety. It could also include a review of what measures are used to assess the accuracy of evidence concerning preventative actions, as well as the creation of additional training and resources to support implementation at service, director, and staff levels.

Collegial, collaborative and mutually respectful relationships, and safe and supportive organisational climates, are essential to ensuring the health and safety of ECEC staff, as well as supporting the provision of high-quality experiences for children. Indeed, many of the protective factors identified in this study relate to a sense of being part of a team with a shared goal. Greater attention and action to prevent workplace bullying are clearly needed by all stakeholders in the ECEC sector.
